# Editorial: Insights in molecular and cellular reproduction

**DOI:** 10.3389/fcell.2026.1912508

**Published:** 2026-07-08

**Authors:** Anthony Valverde, Asok K. Dasmahapatra

**Affiliations:** 1 Laboratory of Animal Reproduction, Research and Development Center for Sustainable Agriculture in the Humid Tropics, School of Agronomy, Costa Rica Institute of Technology, San Carlos Campus, Alajuela, Costa Rica; 2 Environmental Toxicology Division, Department of BioMolecular Science, School of Pharmacy, University of Mississippi, Oxford, MS, United States

**Keywords:** fertility mechanisms, gamete competence, meiotic regulation, mitochondrial metabolism, somatic cell signalingm

## Introduction

Reproduction is sustained by highly coordinated cellular and molecular processes that operate across gametes, somatic support cells, and reproductive tissues (Coticchio et al., 2015). Although reproductive success is often assessed through clinical or laboratory endpoints such as semen quality, oocyte competence, embryo development, or fertility outcomes, these phenotypes arise from complex biological networks involving metabolism, mitochondrial function, meiotic control, genome protection, cell signaling, apoptosis, and intercellular communication (Sutovsky et al., 2015; Voros et al., 2025). The aim of this Research Topic is to bring together studies that advance our understanding of these mechanisms in both physiological and pathological contexts, while placing molecular and cellular reproductive biology within a broader translational framework.

Rather than viewing infertility as the consequence of isolated defects in gametes or reproductive tissues, the articles included collectively support a more integrated view of reproductive competence (Morin and Scott, 2018). Fertility emerges from the coordinated activity of germ cells, somatic support cells, and reproductive microenvironments, all of which must preserve metabolic balance, signaling precision, genome integrity, and cellular resilience (Wang et al., 2024). In this regard, the Research Topic illustrates a transition in reproductive biology from descriptive assessment toward mechanistic, quantitative, and systems-level interpretations of reproductive function. It includes 1 review, and 9 original research articles, 4 of them belong to female fertility/reproduction and 6 of them (5 articles and one review) are on male fertility/reproduction, providing critical and novel insights into the cell and developmental biology.

## Female fertility

Female fertility is strongly associated with several processes. The 4 articles in this Research Topic, introduce and discuss aspects of female fertility due to endometriosis, polycystic ovary syndrome (PCOS), and disorders in gametogenesis and cell division. Disruptions at the microscopic levels frequently result in diminished ovarian reserve, oocyte arrest, or early embryonic loss. Endometriosis is a chronic inflammatory disease condition where tissue similar to the lining of the uterus grows outside the uterus, usually in the pelvis. This misplaced tissue thickens and bleeds with each menstrual cycle, leading to inflammation, severe pelvic pain, and sometimes fertility issues (Smolarz et al., 2021). Although the exact cause of endometriosis has not yet been established, several factors including hormonal, immunological, congenital, environmental, epigenetic, autoimmune, and allergic conditions are considered as a potentially regulator of the disorder.

Zhou et al. further advanced this concept by examining follicular fluid-derived exosomal LINC02701 in active endometriosis. Their study linked altered exosomal lncRNA cargo with granulosa cell apoptosis through the GRP75–P53 axis and associated these cellular changes with impaired early embryo quality. This contribution extends current understanding beyond descriptive exosomal profiling by proposing a mechanistic connection between inflammatory disease activity, extracellular vesicle cargo, granulosa cell injury, and reproductive outcome.

A second theme is the reproductive disease microenvironment, particularly in endometriosis. Hamutoğlu et al. integrated ultrastructural, morphometric, and molecular analyses to examine KiSS-1 localization and PI3K/AKT signaling in endometriotic tissues. Their findings suggest that altered PI3K/AKT activity, nuclear KiSS-1 localization, mitochondrial stress, autophagy-related structures, and stromal remodeling may reflect adaptive responses to chronic inflammation and hypoxia.

Han et al. identified CNOT6L as a regulator of granulosa cell energy metabolism in polycystic ovary syndrome. CNOT6L upregulation inhibited glycolysis, activated mitochondrial oxidative phosphorylation, reduced lactate production, and may thereby impair the energetic support required for oocyte maturation. This work links post-transcriptional regulation, poly(A) tail dynamics, metabolic homeostasis, and follicular dysfunction, offering a mechanistic perspective on granulosa cell involvement in PCOS-associated reproductive impairment.

A fourth theme concerns meiotic fidelity, chromosome segregation, and genome protection. Blengini and Schindler, in this brief report, completed a genetic interaction map of Aurora kinases in mouse oocytes and reinforced the essential role of AURKA in spindle formation, chromosome segregation, and female fertility. Their findings clarify the non-equivalent roles of AURKA, AURKB, and AURKC in oocyte meiosis and highlight the relevance of kinase specialization in preventing aneuploidy. In contrast, Qin et al. showed that germline deletion of Nsmce2 does not impair mouse spermatogenesis or fertility under physiological conditions, despite the known role of NSMCE2 in genome maintenance. This apparent dispensability points to functional redundancy and compensatory mechanisms within the meiotic repair network.

## Male fertility

Male fertility depends on a highly coordinated network of cellular and molecular events, fundamentally governed by spermatogenesis in the testes, molecular maturation, and sperm motility, and the mechanistic of fertilization (Álvarez-Rodríguez and Catalán, 2025). However, male infertility linked to molecular and cellular reproduction often stems from genetic mutations, oxidative stress, and endocrine disruption. These disturbances impair spermatogenesis and sperm motility, directly affects fertilization (Cheng et al., 2025).

Male infertility focused on complex mechanisms related to defects in germ cell development, hormonal regulation, DNA repair, and chromosomal integrity. Other factors, such as transcription factors, structural proteins of the sperm flagellum and proteins involved in meiotic recombination, have increasingly been implicated in conditions ranging from oligozoospermia to non-obstructive azoospermia (Dhikhirullahi and Zhang, 2025). Advances in gene sequencing, single-cell transcriptomics and genome editing have identified genes and pathways, including those modulating cell cycle progression, energy metabolism, and cell-cell communication between SC and germ cells. Moreover, disruptions in androgen signaling and innate immune responses have emerged as contributors to genomic instability in the testis (Su et al., 2024). The 6 articles, including one review article, in this topic, focused on the current state of knowledge, highlights technological breakthroughs and the translational potential of genetic and molecular research in mostly addressing male infertility.

A first major theme is the functional specialization of gametes and the cellular mechanisms that determine fertilization competence. Balestrini et al. demonstrated that SLO3-dependent membrane potential hyperpolarization is required for acrosomal exocytosis and fertilization in the female reproductive tract. Their study distinguishes sperm migration to the ampulla from the acquisition of final fertilizing capacity, showing that sperm transport and sperm-oocyte interaction are not governed by identical requirements. This physiological approach refines our understanding of capacitation by demonstrating that mechanisms inferred from *in vitro* assays require validation in the reproductive tract environment.

Complementing this functional perspective, Skowronek et al. used computational image analysis, fluorescence labeling, and electron microscopy to examine sperm midpiece and mitochondrial morphology in fertile and infertile men. Their study shows how quantitative morphometry can reveal mitochondrial and midpiece alterations that are not fully captured by conventional semen assessment. Together, these contributions demonstrate that sperm quality should not be reduced to motility or morphology alone, but understood through the integration of membrane physiology, organelle structure, energetic competence, and objective analytical pipelines.

Genome regulation is further explored in the review by Hong et al., which synthesizes current knowledge on the piRNA pathway in spermatogenesis and male infertility. The review places PIWI-interacting RNAs at the center of germline genome defense, transposable element silencing, chromatin remodeling, mRNA regulation, and spermatogenic progression. It also discusses the possible use of piRNAs as non-invasive biomarkers and the future potential of piRNA-related pathways in reproductive medicine.

A third theme is the central role of somatic support cells in maintaining gamete competence. Sertoli and granulosa cells are increasingly recognized not as passive structural elements, but as metabolically active regulators of germ-cell development. Falvo et al. showed that D-aspartate directly affects TM4 Sertoli cells by activating ERK/Akt/PCNA signaling, reducing oxidative stress and apoptosis, improving mitochondrial function, and stabilizing mitochondria-associated endoplasmic reticulum membranes.

Fu et al. extended this metabolic concept to obesity-associated male reproductive dysfunction. Using *in vivo* and *in vitro* approaches, they reported that a novel N-salicyloyl tryptamine derivative ameliorated spermatogenic dysfunction in obese mice by improving insulin sensitivity, enhancing Sertoli cell glycolysis, increasing glycolytic enzyme expression, and reducing apoptosis. This study highlights how systemic metabolic disorders may compromise fertility through local disruption of Sertoli cell energy metabolism and lactate support. It also suggests that restoring somatic-cell metabolic function could represent a promising research direction, although further validation is needed before clinical implications can be established.

## Conclusion

Overall, the articles published through this Research Topic show that reproductive competence is an emergent property of interconnected cellular systems. Sperm function depends on membrane potential regulation, mitochondrial organization, and acrosomal responsiveness. Oocyte and spermatogenic competence require meiotic precision, chromosome segregation, DNA repair, and epigenetic regulation. Sertoli and granulosa cells provide essential metabolic and paracrine support, while reproductive pathologies such as obesity, PCOS, and endometriosis disrupt fertility through altered signaling, metabolism, inflammation, apoptosis, and extracellular communication. This Research Topic therefore contributes to a broader view of reproductive biology in which infertility is not merely a clinical endpoint, but the manifestation of disrupted cellular resilience. Ultimately, the studies assembled here reinforce the view that fertility depends on the capacity of gametes, somatic support cells, and reproductive tissues to preserve function under metabolic, genetic, inflammatory, and environmental stress. This Research Topic helps define new directions for reproductive biology, assisted reproduction, fertility preservation, and the diagnosis and management of reproductive disorders.
